# Rapid Actuation of Thermo-Responsive Polymer Networks: Investigation of the Transition Kinetics

**DOI:** 10.1021/acs.jpcb.2c01160

**Published:** 2022-04-14

**Authors:** Simone K. Auer, Stefan Fossati, Yevhenii Morozov, Dario Cattozzo Mor, Ulrich Jonas, Jakub Dostalek

**Affiliations:** † Biosensor Technologies, AIT-Austrian Institute of Technology GmbH, Konrad-Lorenz-Straße 24, Tulln an der Donau 3430, Austria; ‡ CEST Competence Center for Electrochemical Surface Technologies, Tulln an der Donau 3430, Austria; § Macromolecular Chemistry, Department Chemistry-Biology, University of Siegen, Siegen 57076, Germany; ∥ Czech Academy of Sciences, FZU-Institute of Physics, Na Slovance 2, Prague 182 21, Czech Republic

## Abstract

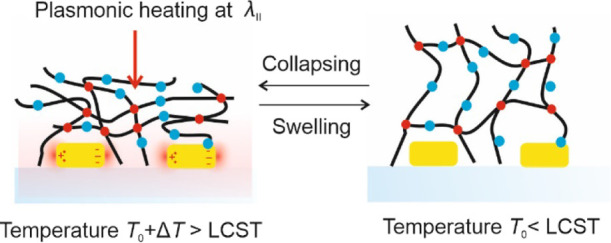

The swelling and collapsing of thermo-responsive poly(*N*-isopropylacrylamide)-based polymer (pNIPAAm) networks are investigated in order to reveal the dependency on their kinetics and maximum possible actuation speed. The pNIPAAm-based network was attached as thin hydrogel film to lithographically prepared gold nanoparticle arrays to exploit their localized surface plasmon resonance (LSPR) for rapid local heating. The same substrate also served for LSPR-based monitoring of the reversible collapsing and swelling of the pNIPAAm network through its pronounced refractive index changes. The obtained data reveal signatures of multiple phases during the volume transition, which are driven by the diffusion of water molecules into and out of the network structure and by polymer chain re-arrangement. For the micrometer-thick hydrogel film in the swollen state, the layer can respond as fast as several milliseconds depending on the strength of the heating optical pulse and on the tuning of the ambient temperature with respect to the lower critical solution temperature of the polymer. Distinct differences in the time constants of swelling and collapse are observed and attributed to the dependence of the cooperative diffusion coefficient of polymer chains on polymer volume fraction. The reported results may provide guidelines for novel miniature actuator designs and micromachines that take advantages of the non-reciprocal temperature-induced volume transitions in thermo-responsive hydrogel materials.

## Introduction

Responsive polymers represent an important class of materials that serve in a broad range of applications spanning from drug delivery,^1^ switchable biocompatible coatings,^2^ and advanced biointerfaces in bioanalytical devices^3,4^ to active materials driving miniature actuators and micromachines.^5,6^ Responsive polymers are designed to change their properties with various stimuli, among which temperature is one of the most commonly used. The majority of thermo-responsive polymers possess a lower critical solution temperature (LCST), below which they exhibit a hydrophilic character, which results in cross-linked polymer networks in an open structure that incorporates high amounts of water molecules. Upon increasing the temperature above the LCST, the polymer chains switch to a hydrophobic state and yield a more compact conformation by expelling water from their proximity. A very prominent example for thermo-responsive LCST polymers is poly(*N*-isopropylacrylamide) (pNIPAAm), but many other materials with such a LCST behavior have been developed over time.^7^

Thermo-responsive polymers have been exploited as polymer brushes or networks forming hydrogels after swelling with water in the form of thin films attached to the surface of solid substrates and colloidal particles^8−10^ or as microgels with spherical^11^ and more complex shapes.^12−15^ One of the key characteristics of devices prepared from LCST hydrogels is the speed, with which they can respond to changes in temperature. For example, surface-attached hydrogel microstructures based on pNIPAAm were developed to actuate microfluidic valves that can be reversibly switched in 5 s.^16^ The same material was employed as thin microgel ribbons^12,17^ to perform optically actuated locomotion by cycling through non-reciprocal morphing in a period of about 1 s. Rapid disassembly of metallic nanoparticle aggregates that are capped with pNIPAAm brushes was reported for mechanical release of energy on a μs time scale via a spring-loaded mechanism.^18^

In general, the response time of actuated thermo-responsive polymer devices with hydrogel network and brush architectures depends on various factors, like the speed of the individual chain response with respect to its solvation state, their collective conformation switching, and on the diffusion of water molecules in and out of the network structure. The latter effect is particularly detrimental with increasing size of the hydrogel structures, and it is worth noting that recently developed hydrogel composite materials allow overcoming this limitation in part, owing to its highly porous structure.^5^

The swelling and collapse kinetics of pNIPAAm-based brushes and networks are commonly investigated by changing the bulk temperature of the aqueous environment comprising the studied specimen.^19^ However, the temperature variation under these conditions is typically slow due to the large heat capacity of the macroscopic volume, which limits the accessible kinetic range. Faster modulation could be achieved by more local heating approaches, for example, based on thin resistive ITO microheaters^4^ or particularly via plasmonic heating.^20^ Plasmonic heating relies on the optical excitation of localized surface plasmon (LSP) modes on the surface of metallic nanostructures, which originate from collective oscillations of the electron density and the associated electromagnetic field. These resonances optically probe the close vicinity of the metallic nanostructure and dissipate to heat via Ohmic losses in the metal. Therefore, metallic nanostructures can serve as efficient optically controlled local heat sources.^21^ In conjunction with thermo-responsive polymers, such plasmonic heaters were implemented by loading microgels from pNIPAAm polymer networks with synthetically made metallic nanorods^13^ or by capping individual metallic nanoparticles with pNIPAAm chains when attached to a substrate^22^ or used in the form of a colloid.^18^

The swelling and collapse kinetics of 30 nm-thick pNIPAAm brushes on lithographically fabricated plasmonic nanoparticles demonstrated a single-exponential behavior on a time scale of 0.16 ms.^20^ Contrary to this, the pNIPAAm polymer network forming a hydrogel film with a thickness of 600 nm revealed more complex swelling and collapse kinetics. The hydrogel layer responded in two phases with the faster component exhibiting a response time below 100 ms and the slower process occurring on a time scale of seconds.^4^ These signatures have been attributed to the effect of water diffusion into and out of the structure and subsequent slower rearrangement and collective motion of interconnected polymer chain segments. The related effect of chain entanglement and other intrachain and interchain interactions was ascribed to the occurrence of hysteresis in the swelling and collapsing process for a pNIPAAm brush upon slowly varying temperature around the LCST.^23^

In this work, we apply rapid plasmonic heating in order to investigate differences in the swelling and collapsing kinetics of pNIPAAm networks on a time scale that was, so far, used only with thin brush architecture.^20^ We further study these transitions as a function of the strength of the local temperature increase, the heating pulse duration, and ambient temperature offset with respect to the LCST of the polymer. The external parameters are controlled to determine the ultimate actuation speed and to devise possible routes for efficient and rapidly operated micromachines and nanomachines.

## Methods

### Preparation of Au Nanoparticle Arrays

A He–Cd laser (IK 3031 R–C from Kimmon, Japan) emitting at λ = 325 nm with 4 mW was employed. The coherent beam was expanded with a spatial filter consisting of a pinhole (10 μm) and ×40 microscope lens. The expanded beam was collimated by using a fused silica lens (*f* = 1 m), and its intensity was measured to be 10 μW cm^–2^. Au nanoparticle arrays were prepared as previously reported by the use of the two-beam laser interference lithography with Lloyd’s configuration.^24^ Briefly, 2 nm-thick Cr and 50 nm-thick Au layers were evaporated on top of BK7 glass slides with a size of 25 × 25 mm (HHV AUTO 306 from HHV Ltd, UK, in a vacuum better than 10^–6^ mBar). Subsequently, a 100 nm-thick layer of S1805 positive photoresist (from micro-resist technology GmbH, diluted 1:2 with propylene glycol monomethyl ether acetate) was spun at a spin rate of 4500 rpm for 45 s. Soft baking of the resist was conducted at 98 °C for 2 min. The angle of the interfering beams in Lloyd’s configuration was set to 21.17°, yielding a period of Λ = 450 nm, and the dose was set to 4.2 and 2.4 J cm^–2^ in the two orthogonal directions. The parameters were adjusted to obtain arrays of resist features exhibiting an elliptical footprint with a distinct short axis length *D*^⊥^ and a long axis length *D*^∥^ after immersing the substrate in the AZ303 developer (1:15 ratio deionized water). Such a resist mask was transferred to the underlying gold layer using a dry etching process consisting of bombardment of the surface with argon ions (Roth & Rau IonSys 500, Germany, 5 × 2 min etching with 2 min pauses in between). Resist-free Au nanoparticles were finally obtained by exposing the substrate to an oxygen plasma process.

### AFM Analysis of Au Nanoparticle Arrays

The topography of the prepared arrays of Au nanoparticles was measured by using atomic force microscopy (PicoPlus from Molecular Imaging, Agilent Technologies, Germany) with tapping-mode tips PPP-NCHR-50 (Nanosensors, Switzerland). The obtained images were processed in open-source software Gwyddion (version 2.47 from gwyddion.net).

### Synthesis of pNIPAAm

The pNIPAAm-based terpolymer was synthesized using *N*-isopropylacrylamide (NIPAAm), methacrylic acid (MAA), and *N*-(4-benzoylphenyl)acrylamide (BPAAm)^25^ (94:5:1 ratio) monomers according to the procedures previously reported.^26,27^ A 50 mL Schlenk flask was charged with 1,4-dioxane (20 mL). Oxygen was then removed by degassing the solvent and flushing with argon. This procedure was repeated three times, followed by the addition of NIPAAm (17.8 mmol, 2.01 g), MAA (1.2 mmol, 0.1 g), and BPAAm (0.18 mmol, 0.045 g). After stirring the solution at room temperature for 30 min, AIBN (0.22 mmol, 0.036 g) was added, and the solution was heated up 60 °C for 18 h. After cooling to room temperature, the resulting polymer was precipitated in ice-cold Et_2_O, filtered off, and washed again with Et_2_O several times. The polymer was isolated by drying at 50 °C under reduced pressure, and the yield was 77% (1.66 g).

### Preparation of the pNIPAAm-Based Hydrogel Layer

In order to attach the hydrogel to the Au nanoparticle arrays, their surface was modified with benzophenone moieties by overnight reaction with 1 mM benzophenone disulfide (synthesized as described before^25^) dissolved in DMSO. Subsequently, a pNIPAAm-based polymer dissolved in ethanol (2 wt %) was spun (2000 rpm for 2 min) over the Au nanoparticle array surface followed by drying overnight in a vacuum (*T* = 50 °C). The polymer layer was then simultaneously cross-linked and attached to the surface by irradiation with UV light (dose of 2 J/cm^2^ at λ = 365 nm). The thickness of the hydrogel layer attached to the Au nanoparticles in a dry and swollen state was measured using an in-house sensor system based on surface plasmon resonance (SPR) (see Supporting Information, part 1).

### Optical Setup for LSPR Tracking and Plasmonic Heating

**Figure 1:**
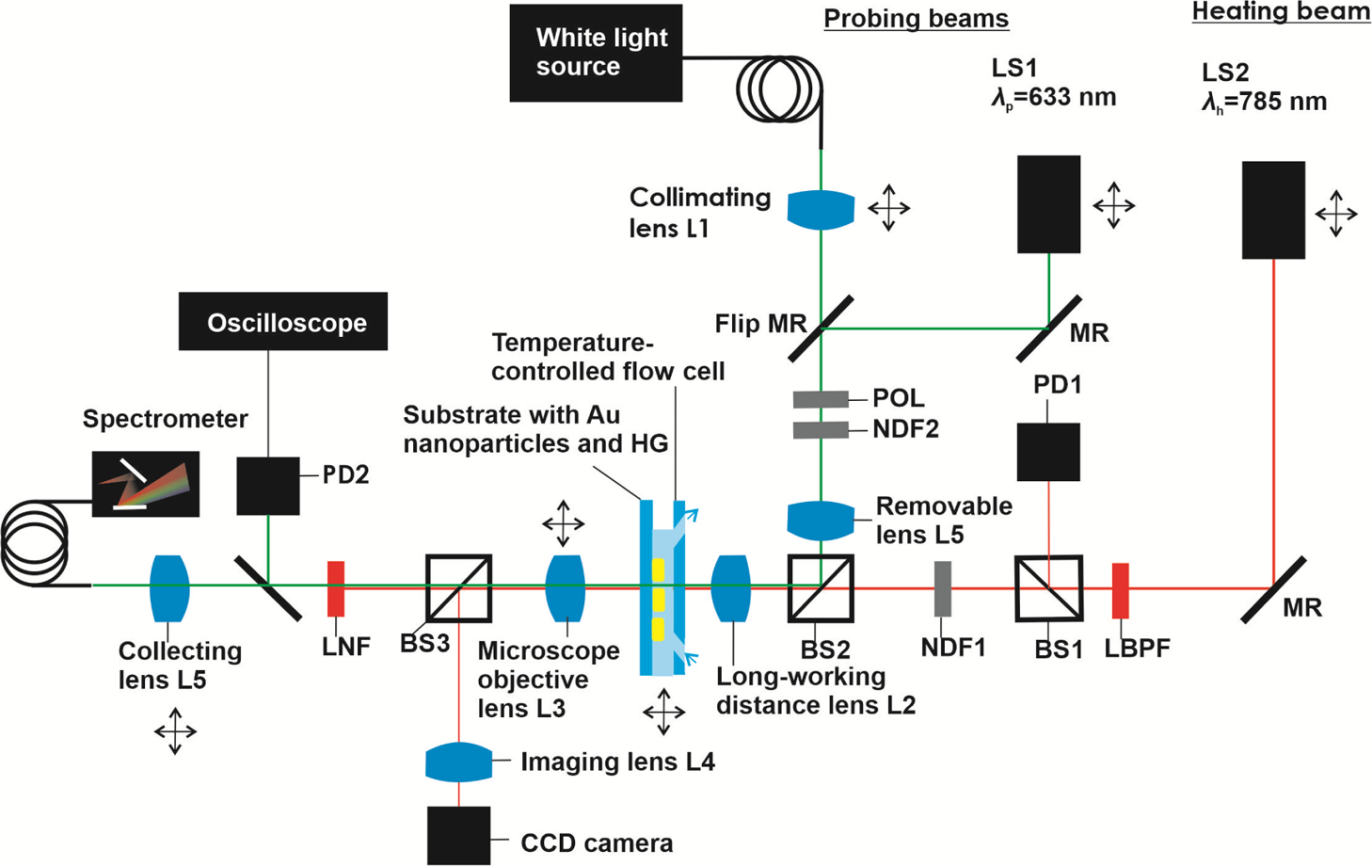
Optical setup employed for the combined wide-field optical microscopy observation of the surface carrying arrays of Au nanoparticles with a thermo-responsive hydrogel (HG) overlayer and for the beam alignment, transmission wavelength spectroscopy-based recording of LSPR spectra, and for the optical heating and monitoring of rapid LSPR kinetics.

An in-house developed optical instrument is schematically shown in Figure 1. The glass substrate with arrays of Au nanoparticles and a pNIPAAm-based hydrogel layer on the top was clamped to a flow cell. The flow cell chamber was formed by using a PDMS gasket and a transparent glass lid, which was contacted with a Peltier device in order to control the ambient bulk temperature *T*_0_ (operated with a temperature controller, wavelength electronics, model LFI-3751, USA). The flow cell with the clamped sample carrying the pNIPAAm-based hydrogel layer mounted on a set of XYZ linear stages was optically probed with a series of beams that passed through the fixed long-working distance objective lens L2 (Mitutoyo MPlan Apo 50*x*/0.55, ∞/0 *f* = 200, Japan). The polychromatic beam emitted from a halogen lamp (LSH102 LOT-Oriel, Germany) was coupled to an optical fiber (Thorlabs, UK), delivered to a collimator L1 (Thorlabs, UK), and made to pass through a polarizer POL (B.Halle, Germany). A monochromatic beam at a probing wavelength of λ_p_ = 633 nm emitted from a HeNe laser (LS1, Melles Griot stabilized HeNe Laser system 633 nm, USA) was used, and a monochromatic beam at a different heating wavelength of λ_h_ = 785 nm (LS2, Toptica Photonics iBeam smart WS wavelength-stabilized diode laser 785 nm, 250 mW, Germany) was introduced into the system. The intensity of the heating beam *I*_h_ was chopped, made to pass through a band pass filter LBPF (FB780-10, Thorlabs, United States), and by using a splitter BS1, its intensity was monitored using a photodiode PD1. The polychromatic and monochromatic beams were merged by using a splitter BS2 and launched at the substrate with the pNIPAAm-based layer in the temperature-controlled flow cell. The transmitted beams were recollimated with a lens L3. By using a splitter BS3 (splitting rate 90:10, Thorlabs, UK) and lens L4 (Schneider Kreuznach Unifoc Component 4/35, FL 35, Germany), the surface of the pNPAAm-based layer was imaged using a CCD camera (piA1000-48ag, Basler AG, Germany). The beams that passed through the other arm of BS3 were spectrally filtered with a notch filter LNF (NF785-33, Thorlabs, UK) and were delivered either to a photodiode PD2 (probing beam at λ_p_) or collected by using a lens L5 (FL 90) and launched to an optical fiber (Ocean Optics, USA) and analyzed using a spectrometer (Andor Technology, Shamrock 303 Spectrometer, UK) (polychromatic beam). The intensity of the monochromatic probing beam at λ_p_ was analyzed by using an oscilloscope (Agilent Technologies, DSO 1004A 60 MHz 2GSa/s, United States), where the signal from PD1 (intensity of heating beam at λ_h_) served as a trigger and PD2 (intensity of the probing beam at λ_p_) as an input. The long-distance working lens L2 was used to focus the polychromatic and monochromatic beams on the pNIPAAm-based layer surface. The focus was checked with the CCD camera and the circular areas irradiated with the beams were aligned to overlap and set to the same diameter of 30 μm. For the (static) measurement of LSPR, the spectra acquired by using a polychromatic beam and spectrometer were normalized with those measured on an area of the substrate that did not carry the Au nanoparticle arrays. The measurement of the swelling and collapsing kinetics was performed by using an oscilloscope configuration, and the averaging of the sensorgrams by a factor of 256 was used. The flow cell was connected to a peristaltic pump (Ismatec, Switzerland) in order to transport water across the surface of the pNIPAAm-based hydrogel layer.

### Numerical Simulations

LSPR transmission spectra were simulated with the finite-difference time-domain method (Lumerical Inc., Canada). Following geometrical parameters for arrays of gold nanoparticles were used: an elliptical footprint with a short axis of *D*^⊥^ = 200 nm, a long axis of *D*^∥^ = 260 nm, and a height of 50 nm arranged in rectangular arrays with a period of Λ = 450 nm. Below the gold nanoparticles, a Cr layer of 2 nm was assumed. The BK7 glass interface (with a refractive index of 1.5) was carrying a dielectric layer with a thickness of 845 nm and refractive index *n*_h_ = 1.35 (representing the pNIPAAm-based hydrogel in the swollen state) or a thickness of 105 nm and refractive index *n*_h_ = 1.42 (representing the pNIPAAm-based hydrogel in the collapsed state). As a superstrate, water medium with a refractive index of 1.33 was set. A plane wave made normally incident on the structure was used as an excitation source. Periodic (along the *x*- and *y*-axis) and perfectly matched layer absorbing (on the *z*-axis 1.2 μm above and below the structure) boundary conditions were applied. Au and Cr optical properties were taken from the literature.^28,29^

## Results and Discussion

**Figure 2:**
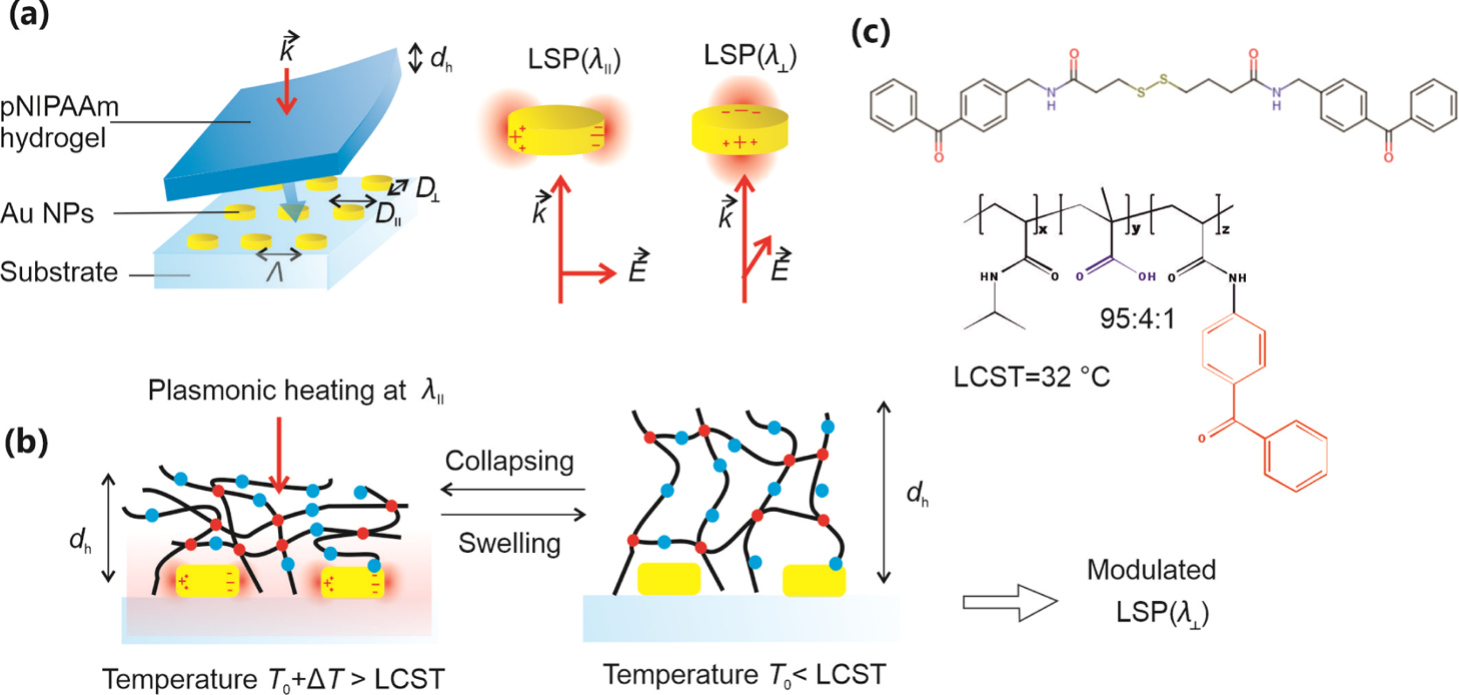
Schematics of the (a) substrate with arrays of elliptical Au nanoparticles that exhibit distinct LSPR wavelengths and that carry thermo-responsive poly(*N*-isopropylacrylamide)-based (pNIPAAm) hydrogel layers on the top. (b) pNIPAAm polymer network swelling and collapsing by using the temperature stimulus and (c) chemical structure of the benzophenone disulfide linker and used pNIPAAm-based terpolymer.

Rapid actuation of pNIPAAm-based, thermo-responsive polymer networks swollen with water was achieved by plasmonic heating of metallic nanostructures. As schematically shown in Figure 2a, these metallic nanostructures were attached to the glass substrate and a layer of the pNIPAAm-based hydrogel was anchored to their top. In order to simultaneously employ the metallic nanostructure for rapid actuation of the thermo-responsive network and for the measurement of its swelling and collapse kinetics, periodic arrays of Au nanoparticles with an elliptical footprint were prepared. On these nanoparticles, LSPs at distinct resonant wavelengths of λ^⊥^ nm and λ^∥^ can be excited when rotating the polarization of the incident beam perpendicular ⊥ or parallel ∥ to their long axis. The LSPs at shorter wavelength λ^⊥^ were then used for the probing of refractive index changes that accompany the swelling and collapse of the pNIPAAm-based polymer network. The LSPs at longer wavelength λ^∥^ were utilized for the coupling of a heating beam and subsequent local dissipation of its energy via the Ohmic losses (see Figure 2b).

### Preparation of pNIPAAm Networks on the Plasmonic Substrate

**Figure 3:**
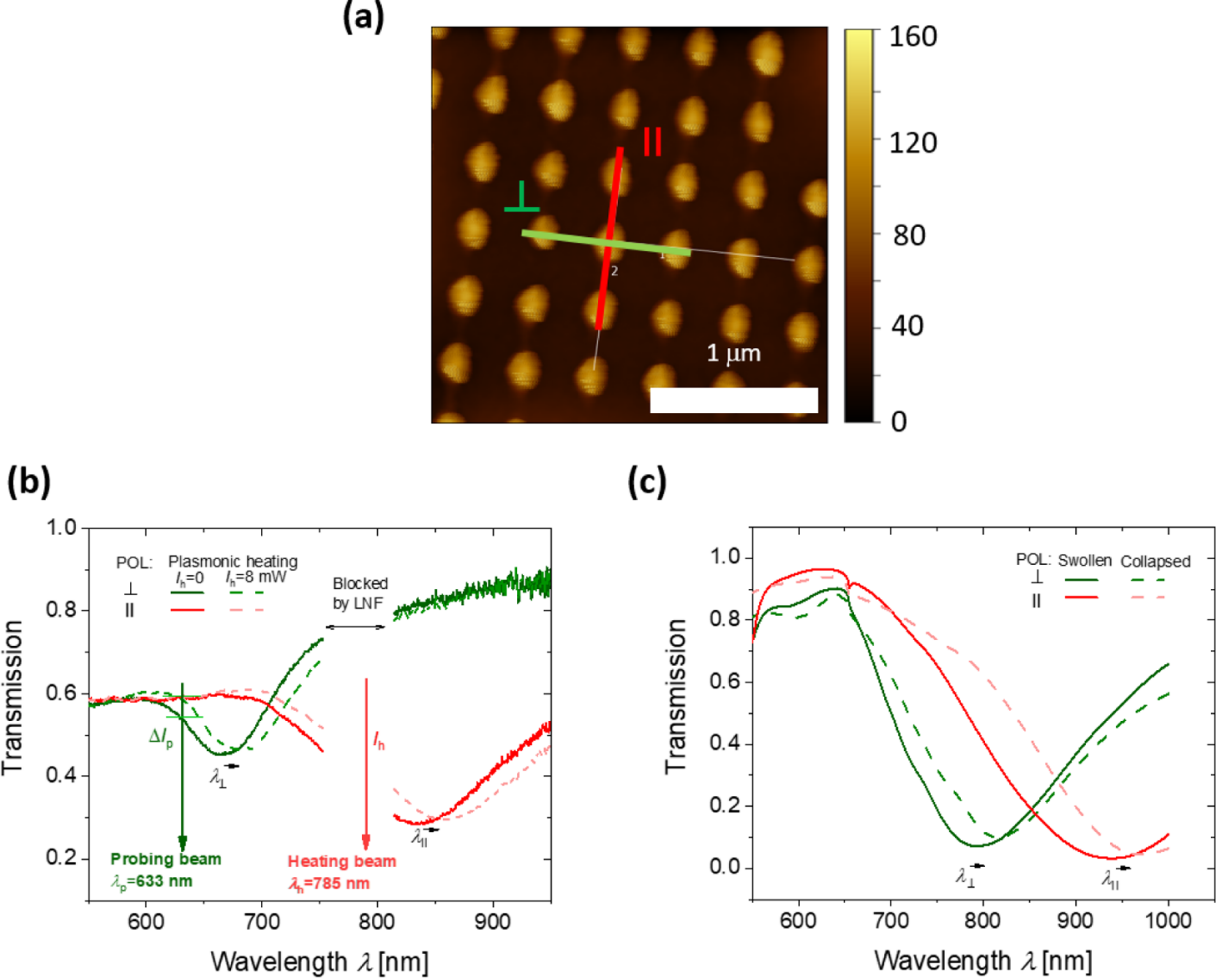
(a) Topography of the prepared Au nanoparticle arrays and comparison of (b) measured and (c) simulated LSPR transmission spectra when the attached pNIPAAm-based hydrogel layer is swollen and collapsed (assuming the refractive index of *n*_h_ = 1.35 and 1.48, respectively).

Periodic arrays of elliptical Au nanoparticles were prepared by using UV laser interference lithography in combination with a dry etching step. As can be seen from the AFM micrographs in Figure 3a, they exhibited a short axis length of *D*^⊥^= (200 ± 10) nm, a long axis length of *D*^∥^= (260 ± 20) nm, a height of *h* = 50 nm, and the period was set to Λ = 450 nm. For these parameters, the spectral position of LSPR bands could be tuned close to the heating beam wavelength (λ_h_ = 785 nm) and probing beam wavelength (λ_p_ = 633 nm). The surface of the Au nanoparticles was chemically modified with a self-assembled monolayer of disulfide molecules with a photo-reactive benzophenone headgroup (Figure 2c). The pNIPAAm-based polymer layer was deposited on top with a (dry) thickness of *d*_h_ = 104 nm. The terpolymer carried a small amount of negatively charged methacrylic acid groups promoting swelling. In addition, photo-reactive benzophenone groups served for simultaneous cross-linking and attaching to Au nanoparticles upon irradiation with UV light. When the UV-cross-linked layer was contacted with water, the polymer network took up water molecules, leading to its swelling and increase in the thickness to *d*_h_ = 843 nm at room temperature *T*_0_ = 24 °C. Surface plasmon resonance combined with optical waveguide spectroscopy (see Supporting Information S1) showed that the pNIPAAm-based hydrogel exhibits a refractive index of *n*_h_ = 1.48 when it is dry and decreases to 1.35 when it swells in water. By raising temperature above LCST to 40 °C, the thickness decreases by a factor of 4.7 and the refractive index increases to *n*_h_ = 1.42.^30^

### LSPR Probing of the pNIPAAm Layer

After the attachment of the pNIPAAm-based polymer network atop the Au nanoparticle arrays, the substrate was clamped against a flow cell and water was transported over its surface. As can be seen in Figure 3b, the excitation of the LSPs with their dipole moment aligned along the short and long axes manifests itself as two spectrally distinct dips in the measured wavelength transmission spectrum. For the polarization of the incident optical beam aligned perpendicular to the long axis, the LSPR occurs at a wavelength of about λ^⊥^ = 650 nm, when the pNIPAAm-based hydrogel layer is kept at room temperature. When the polarization is rotated by 90° to align with the long axis of the elliptical nanoparticles, the LSPR is switched to about λ^∥^ = 850 nm. This LSPR dip was tuned close to the wavelength of the near-infrared heating beam (λ_h_ = 785 nm). When irradiating the surface with the heating beam power of *I*_h_ = 8 mW, both LSPR dips shift to longer wavelengths due to the induced collapse of the pNIPAAm-based hydrogel that results in the compacting of the thermo-responsive polymer network on the nanoparticle surface. It leads to an increase in the polymer volume fraction Φ, and thus, it is associated with a proportional increase in the refractive index *n*_h_ (see also Supporting Information section 7). This effect is ascribed to the local change in temperate Δ*T* that exceeds LCST of the pNIPAAm polymer network. In order to relate the spectral shifts of LSPR bands to variations in refractive index *n*_h_ and thickness *d*_h_ of the pNIPAAm-based polymer network film, numerical simulations were carried out. Data presented in Figure 3c predict the shift in the LSPR wavelength by about Δλ^⊥^/Δ*n*_h_ = 183 nm/RIU and Δλ^∥^/Δ*n*_h_ = 238 nm/RIU. Based on these values, the measured spectral shifts of Δλ^⊥^ = 20 nm and Δλ^⊥^ = 26.1 nm correspond to a change in the refractive index of the film of Δ*n*_h_ = 0.11 RIU. This value translates to the swelling ratio change of a flat film of 7.5, which is in the range of the one measured previously for the same type of polymer and cross-linking.^30^ It is worth of noting that the simulated LSPR spectra qualitatively agree with the measured ones, but the LSPR wavelengths are red-shifted with respect to the measured ones by about 150 nm. This discrepancy can be ascribed to the over-estimation of Au nanoparticle short- and long-axis lengths *D*^⊥^ and *D*^∥^ due to the convolution of tip geometry of the used AFM tip with the particle geometry.

Let us point out that the herein-measured LSPR shifts due to temperature changes can be dominantly attributed to swelling and collapsing of the investigated pNIPAAm-based hydrogel. Other effects are significantly less pronounced and are associated with optical changes at least one order of magnitude weaker. As discussed in Supporting Information in detail (Section 2), these effects include thermal expansion of the substrate, modulating the period of the gold nanoparticle arrays Λ, and temperature variations of the refractive index of the glass substrate and water.

### Calibration of Plasmonic Heating-Induced Local Temperature Changes

**Figure 4:**
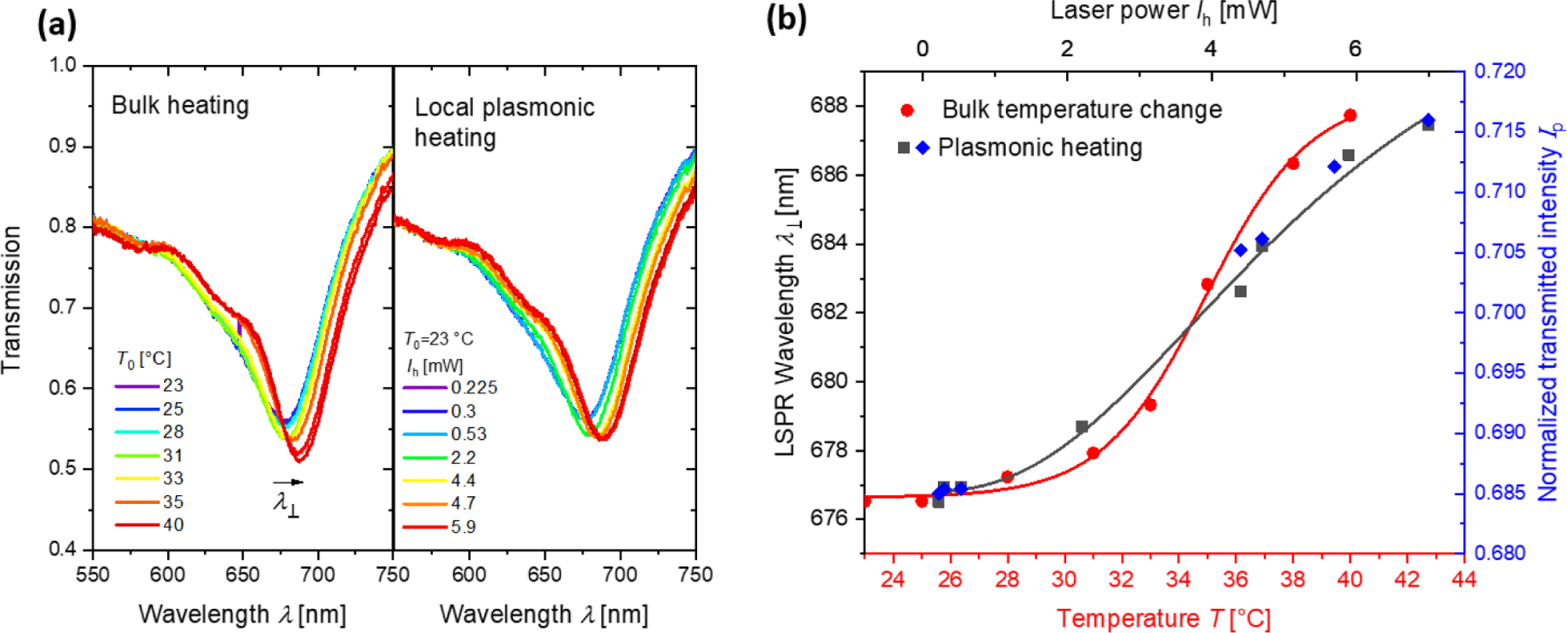
(a) LSPR spectra centered at λ^⊥^ measured for variable bulk temperature *T*_0_ and the same LSPR spectra acquired for increasing intensity of the heating beam. (b) Comparison of the LSPR shifts, enabling calibration of the temperature changes due to the plasmonic heating. The measured data are shown as symbols, and lines are sigmoid function fits that are overlaid to match the response at *I*_h_ = 0 and at the inflection point.

The developed optical system was calibrated by relating the heating beam intensity *I*_h_ (that was focused on a circular area with a diameter of 30 μm) to the local increase in temperature Δ*T*. Figure 4a compares shifts of the resonant wavelength λ^⊥^ in LSPR transmission spectra caused by a change in the bulk ambient temperature *T*_0_ (induced by a Peltier device) with those locally induced (through the effect of plasmonic heating) by gradually increasing the continuous irradiation power *I*_h_ of the heating beam from 0 to 5.9 mW. Both graphs show a red shift of the LSPR absorption band of similar magnitude that is ascribed to the gradual collapse of the pNIPAAm-based hydrogel. Assuming that the effect of the temperature-induced decrease in the refractive index of water and potential changes in the period of the nanoparticle arrays due to thermal expansion can be omitted, the thermally and irradiation-induced LSPR wavelength shifts are related in Figure 4b. The measured data were fitted with sigmoid function and overlaid to match the response at *I*_h_ = 0 and at the inflection point. This relationship is used to convert the heating beam irradiation power at λ_h_ to the local temperature increase on the surface of the nanoparticles Δ*T* in order to show which applied laser powers *I*_h_ correlate with specific temperature changes Δ*T*. For the maximum used power of *I*_h_ = 7 mW, a temperature increase as high as Δ*T* = 15 K was determined. It should be noted that the dependence of the LSPR wavelength λ^⊥^ on the bulk temperature *T*_0_ exhibits more abrupt changes in the vicinity to LCST than that measured with the plasmonic heating. This can be attributed to the possible gradient in the local temperature increase Δ*T* due to the Gaussian profile of the heating beam intensity irradiating a footprint with a diameter of 30 μm.

Moreover, the monitoring of swelling and collapse of the pNPAAm-based polymer network was utilized by probing the LSPR changes at a fixed wavelength of λ_p_ = 633 nm. Then, the red shift of the LSPR band was manifested as an increase in the transmitted intensity of the beam *I*_p_ (see Figure 3b). As it follows the same trend as the variations in resonant wavelength λ^⊥^ (see right axis in Figure 4b), it was used for the monitoring of the fast actuation of pNIPAAm hydrogel by using an oscilloscope at the output.

### pNIPAAm Network Swelling and Collapsing Kinetics

**Figure 5:**
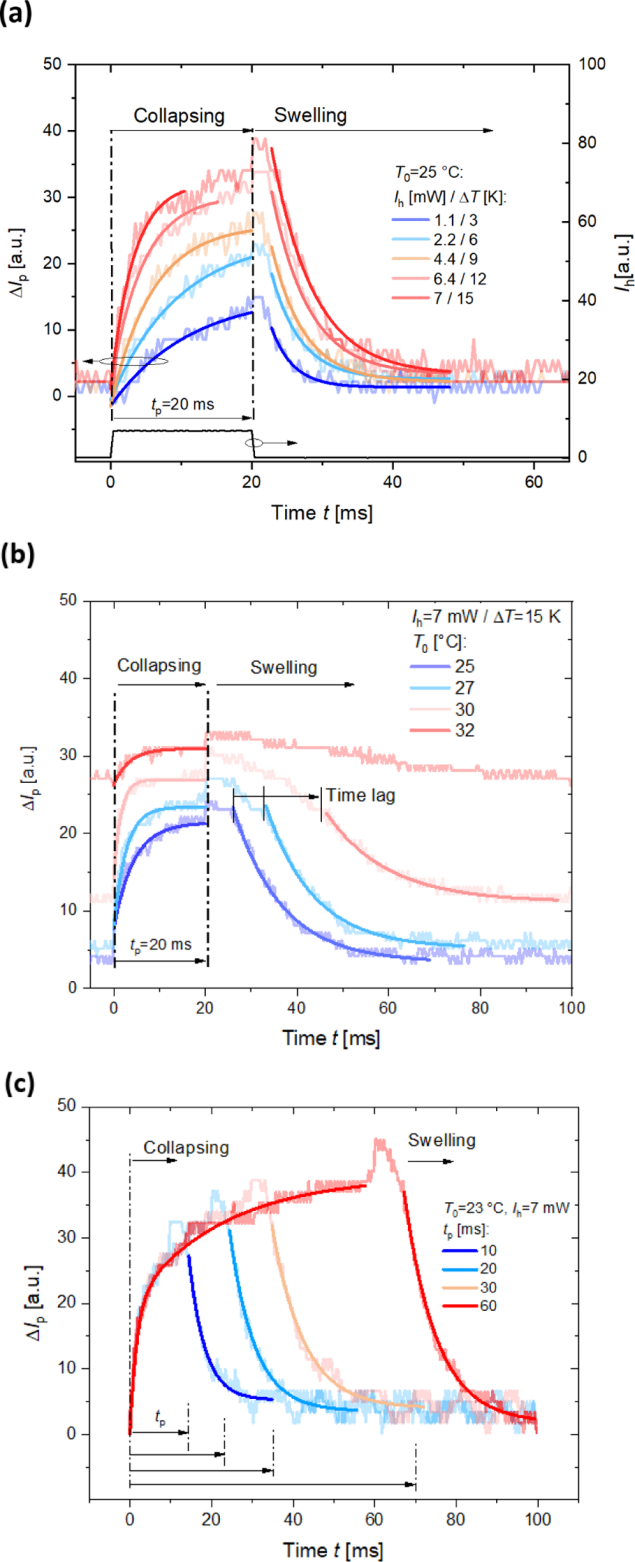
Recorded LSPR kinetics for the pNIPAAm-based polymer network collapse and swelling when (a) varying local temperature increase Δ*T* from below to above LCST, (b) increasing the bulk temperature *T*_0_ to LCST and applying constant Δ*T*, and (c) for changing the time duration of the plasmonic heating pulse *t*_p_. The measured data (presented as curve with light color) are fitted with exponential functions (shown as a curve with corresponding dark color).

In order to measure the kinetics of the swelling and collapse of the pNIPAAm-based hydrogel layer, the developed optical system was configured for the measurement of LSPR changes at λ_p_ with an oscilloscope, upon applying a series of short pulses of the increased heating beam intensity *I*_h_. Let us note that the temperature changes Δ*T* follow the variations in the irradiation beam intensity *I*_h_ faster than millisecond, as can be seen from the quick decrease in *I*_p_ upon switching on the heating beam (*t* = 0 in Figure 5a,b) and an increase in *I*_p_ when the beam is switched off (*t* = 20 in Figure 5a,b). These abrupt changes can be ascribed to rapid variations in the refractive index of water, which follows the opposite trend compared to that of the investigated pNIPAAm-based film. Similar response time can be estimated from theory when the used plasmonic heating with arrays of gold nanoparticles is in a collective heating regime^31^ (see Section 2 in Supporting Information). After this initial phase, switching “on” the heating beam power *I*_h_ received by the sample at λ_h_ was accompanied with a gradual increase in the probing beam intensity *I*_p_ at λ_p_. When the heating beam is set “off”, a gradual decrease to original baseline occurs. These changes follow the collapse and swelling of the pNIPAAm-based polymer networks through the induced refractive index variations Δ*n*_h_ that are associated with changes in the polymer volume fraction Φ and probed by the LSP field confined on the surface of the Au nanoparticle arrays. Furthermore, considering the large thermal penetration depth into the medium within the characteristic time constant (see Supporting Information, section 4), we assume uniform temperature across the hydrogel layer.

In the first experiment, the bulk temperature of the substrate was set below the LCST to *T*_0_ = 25 °C and the heating pulse strength was set to induce a local temperature increase of Δ*T* = 3, 6, 9, 12 and 15 K for a pulse duration of *t*_p_ = 20 ms. As can be seen in Figure 5a, the collapsing of the network is manifested as a gradual increase in the network density (monitored from LSPR changes associated with respective refractive index changes δ*n*_h_). Particularly, for the higher heating strength, the signal intensity kinetics slows down corresponding to approaching to the equilibrium. The collapsing speed increases with the heating strength (time constants of 9.6 and 3.3 ms were fitted with an exponential function for Δ*T* = 3 and 15 K, respectively). Contrary to the collapsing, the time dependence of the swelling phase does not change significantly with Δ*T* and can be well-fitted with an exponential function time constant of 4.8 ± 1 ms.

In the second experiment, the local heating strength was set fixed to the value of Δ*T* = 15 K and the bulk temperature was increased toward the LCST by setting *T*_0_ = 25, 27, 30, and 32 °C for the pulse duration of *t*_p_ = 20 ms. The acquired response kinetics are presented in Figure 5b, and the collapsing phase reveals that when *T*_0_ approaches the LCST, a faster compaction of the pNIPAAm-based hydrogel film occurs (shorter time constant of 1.5 ms was measured for *T*_0_ = 30 °C). Interestingly, the subsequent swelling after turning off the heating beam shows a more complex behavior and it strongly slows down when *T*_0_ approaches the LCST. Apparently, it exhibits kinetics consisting of two distinct phases. In the initial transition phase (occurring after the switching the local heating “off”), a slow monotonous swelling occurs. After a certain time lag, the kinetics flips to a faster exponential dependency (with a time constant of 12 ± 1.7 ms). The transition phase occurs over the time span that prolongs when *T*_0_ becomes close to the LCST (e.g., can be estimated as 25 ms for *T*_0_ = 30 °C).

In the third experiment, the bulk temperature was adjusted below the LCST to *T*_0_ = 23 °C, the local temperature increase was set to Δ*T* = 15 K, and the heating pulse duration was increased from *t*_p_ = 10 to 60 ms. The measured data in Figure 5c reveal that the collapsing kinetics can be fitted with a single exponential function (with a time constant of 6 ms) for short pulse length *t*_p_ up to 10–20 ms; however, above this time, a clear deviation occurs. For long heating pulses, the collapsing kinetics can be fitted as two overlaid exponential processes with time constants of 1.6 and 23.0 ms (see Supporting Information, section 3).

**Figure 6:**
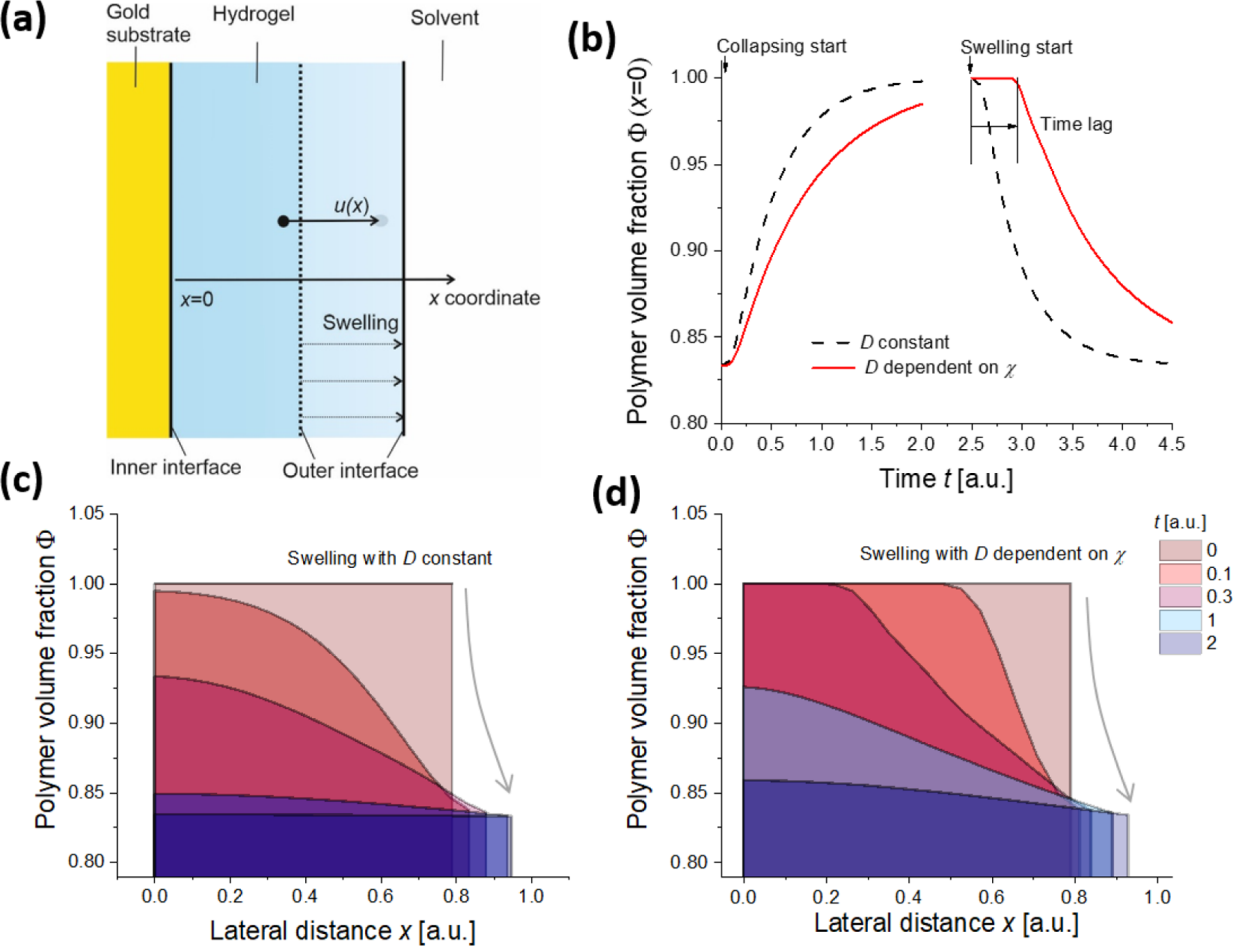
(a) Assumed geometry and definition of the displacement distance describing the swelling and collapsing process of an attached hydrogel layer. (b) Simulated time dependence of the polymer volume fraction Φ upon the collapsing and swelling process at the inner interface (*x* = 0). The spatial distribution of the polymer volume fraction upon the swelling process for the model with (c) constant *D* and (d) when taking into account its dependence on the Flory–Huggins parameter χ.

In order to elucidate the kinetics of the swelling and collapse of responsive hydrogels, a model based on the collective diffusion of polymer chains is often used that was originally introduced by Tanaka et al. for responsive microgels^32^ and later also adopted for other similar systems.^33,34^ As illustrated in Figure 6a, the volumetric changes can be then described with a time-dependent displacement distance *u*(*x*, *t*) for the investigated geometry of a thin layer that is allowed to swell only in the perpendicular direction to its surface and its changes follow the partial differential Fick-like diffusion equation
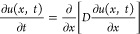
1where *D* is the collective diffusion coefficient. This coefficient is typically assumed to be constant and can be experimentally measured from dynamic light scattering experiments^35^ or theoretically derived as a ratio of osmotic bulk modulus *K* and friction coefficient *f*.^33^ Let us note that such picture holds only for small relative changes in *u*(*x*, *t*) and that a range of effects taking place in the hydrogel layer is omitted including the dynamically changing inhomogeneities,^35^ lateral stress leading to buckling effects,^36^ and the impact of the attachment of the hydrogel to the arrays of Au nanoparticles that perturbed the (assumed) flat geometry.

In order to qualitatively explain some of the experimentally observed changes in the swelling and collapse when modulating the magnitude of the temperature stimulus Δ*T* and varying the ambient temperature *T*_0_, let us take into account the dependence of the collective diffusion coefficient *D* on the Flory–Huggins parameter χ.^34^ This parameter describes that the miscibility of the polymer with the solvent, here water, and for the studied thermo-responsive hydrogel is a function of temperature *T*.^37^ The dependence of *D* on χ can be introduced by using the osmotic pressure Π description of Flory-like mean-field mixing energy with *K* = Φ·∂Π/∂Φ,^33^ (see Supporting Information, part 5) yielding a form of
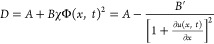
2where *A*, *B,* and *B*′ are constants. Then, the diffusion coefficient is described as a function of the *x* coordinate and decreases (and thus slows down the swelling and collapse process) when the polymer volume fraction Φ increases. In addition, the thermo-responsiveness of the material can be captured based on the dependence of the Flory–Huggins parameter χ, enabling switching between a good solvent, that is, low χ, and a poor solvent, that is, high χ, regime.

Furthermore, let us analyze the time lag that was experimentally observed for the swelling process and that was absent in the collapsing process after the external stimulus was triggered [see Figure 5b]. By numerically solving the above-described partial differential eq 1 with appropriate boundary and initial conditions (see Supporting Information, part 6), we modeled the kinetics of the polymer volume fraction Φ(*x*, *t*) for the swelling and collapse and explored the impact of the dependence of collective diffusion coefficient *D* in form of eq 2. Let us note that then these simulations presented in Figure 6b,c can be related to the experimental data shown in Figure 5a–c as changes in Φ are directly proportional to the refractive index variations δ*n*_h_ measured by LSPR at the inner interface of the hydrogel film (where *x* = 0).

As seen in the simulations results presented in Figure 6b, the swelling and collapse processes are predicted to show a similar exponential behavior when the collective diffusion coefficient *D* is set constant. However, the introduced dependence of *D* on the polymer volume fraction Φ in form of eq 2 leads to a different character of the swelling and collapse kinetics that can be ascribed to the effective decreasing of *D* in the zones with high Φ, which slows down both swelling and collapse processes in these regions. The collapse time dependence shows an exponential behavior as it occurs from the initially swollen hydrogel, which thus exhibits low Φ, and a corresponding high collective diffusion coefficient *D*. The reverse swelling process is different; it starts from compact collapsed hydrogel with low *D,* and the respective time dependence of the polymer volume fraction at the inner interface Φ(*x* = 0, *t*) is more complex and exhibits a pronounced delay with respect to the stimulus time. This behavior qualitatively agrees with the experimental observations in Figure 5b and suggests that for the studied system with strong changes in the swelling ratio, the effect of dependence of the collective diffusion coefficient on Φ cannot be omitted.

The origin of the delayed swelling process is illustrated by simulating the hydrogel density profile during the swelling process. The data presented in Figure 6c,d show the re-distribution of the polymer volume fraction Φ in the gel layer as a function of the distance from the surface *x* and time *t*. They predict that for the constant *D*, the swelling process is associated with the Gaussian distribution of polymer volume fraction Φ(*x*) that rapidly changes with time through the whole layer. However, the proposed modification of *D* with introduced dependence on the Flory–Huggins parameter χ leads to slowing down of the swelling propagation from the outer to inner hydrogel interfaces. This effect leads to the pronounced offset in the swelling at the inner interface, which is not present for the collapse.

## Conclusions

The obtained results reveal that the speed of swelling and collapsing of thermo-responsive polymer networks can, besides design of its chemical structure, be to a large extent controlled by the external parameters. As herein investigated for a micrometer-thick pNIPAAm hydrogel layer that is attached to a solid substrate, the response time of this network to an abrupt change in temperature (controlled on a time scale < 1 ms) substantially differs for the swelling and collapsing phases. The collapsing time scales with the difference between LCST and triggering (increased) temperature change and reaches a value as low as 1.5 ms. Interestingly, when the temperature pulse is prolonged, an additional slower collapsing transition with a characteristic time > 20 ms is overlaid, which has not been measured by a similar technique on non-cross-linked pNIPAAm polymer brushes.^22^ The opposite transition from the collapsed to the swollen state is less affected by the magnitude of driving temperature change, and it occurs in two consecutive regimes. A time lag exceeding 20 ms is seen before the exponential transition to the swollen state proceeds with a time constant close to 10 ms. Based on the performed simulations, the time lag can be attributed to a potential gradient in the film that is due to the initial swelling at the outer interface (in contact with the solvent) propagating toward the optically probed inner interface (on the surface of the solid substrate). In general, these features indicate that the swelling and collapsing of small responsive hydrogel objects is a complex process, and among others, it can provide advantage in design of recently emerged optically driven soft actuators and machines. Their considering may be important for systems researched to perform non-reciprocal morphing as a prerequisite for locomotion of soft micro-swimmers,^12^ time-dependent switching between the hydrophobic and hydrophilic state can be utilized for construction of micro-crawlers,^6^ or in design of responsive biointerfaces that allow rapid compacting of molecular species at plasmonic hotspots for subsequent sensitive plasmonically enhanced fluorescence spectroscopy detection.^30^

## Supporting Information Available

Calculation of the surface mass density of the hydrogel layers (PDF)

## Abbreviations

LSPR: localized surface plasmon resonance
pNIPAAm: poly(*N*-isopropylacrylamide)
LCST: lower critical solution temperature;
