# Supplementary material for: Rapid Actuation of Thermo-Responsive Polymer Networks: Investigation of the Transition Kinetics
Source: J Phys Chem B. 2022 Apr 14;126(16):3170–9. doi: 10.1021/acs.jpcb.2c01160 (PMC9059119; doi:10.1021/acs.jpcb.2c01160)
Supplement: Supplementary file 1 — jp2c01160_si_001.pdf [file jp2c01160_si_001.pdf]

Supporting information to:

Rapid Actuation of Thermo-Responsive Polymer Networks: Investigation of the Transition Kinetics

Simone K. Auer,[† ‖] Stefan Fossati,[†] Yevhenii Morozov,[†] Dario Cattozzo Mor,[§] Ulrich Jonas,[‡] Jakub Dostalek[† § *]

† Biosensor Technologies, AIT-Austrian Institute of Technology GmbH, Konrad-Lorenz-Straße 24, 3430 Tulln an der Donau, Austria

‖ CEST Competence Center for Electrochemical Surface Technologies, 3430 Tulln an der Donau, Austria

‡ Macromolecular Chemistry, Department Chemistry-Biology, University of Siegen, Siegen 57076, Germany

§ FZU-Institute of Physics, Czech Academy of Sciences, Na Slovance 2, Prague 182 21, Czech Republic

1. Characterization of thin pNIPAAm-based layer

Surface plasmon resonance (SPR) measurements were combined with optical waveguide spectroscopy and performed in order to determine the thickness of used pNIPAAm-based film. It was attached to a glass substrate with a 50 nm thick gold layer and an optical instrument with Kretschmann configuration of attenuated total internal reflection geometry was used with a 90° prism made of LASFN9 glass. Angular reflectivity spectra $R(\theta)$ presented in Figure S1 were measured at the wavelength of 633 nm. They were fitted with the Fresnel reflectivity model and the polymer layer thickness of 104 nm was determined for the dry state and of 843 nm for the swollen state at room temperature. The fitting was performed by using a software Winspall developed at Max Planck Institute of Polymer Research in Mainz, Germany.

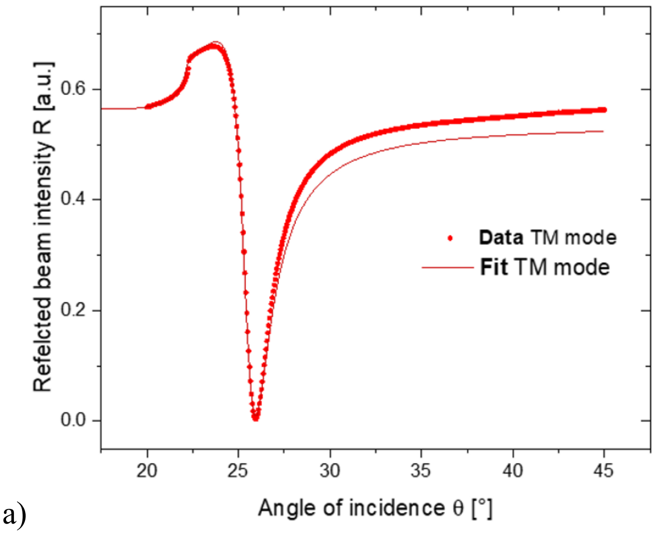

a)

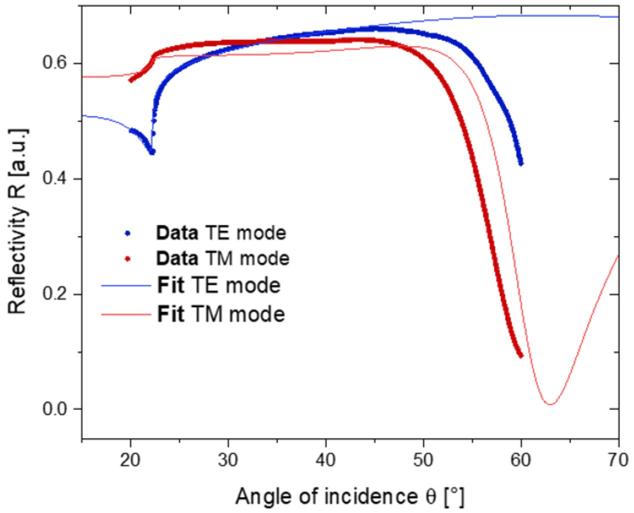

b)

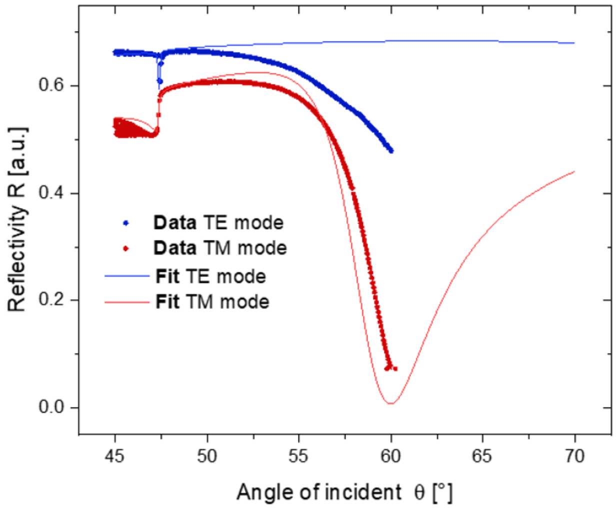

c)

Figure S1 Measured SPR spectra with a respective fit for a) gold-coated substrate, b) gold-coated substrate carrying dry pNIPAAm-based film, c) gold-coated film carrying swollen pNIPAAm-based polymer network in water at room temperature.

2. LSPR changes due to temperature changes: comparison

The refractive index change upon full collapsing the used pNIPAAm-based hydrogel was measured as of Δn_h=0.11 when increasing temperature by ~ 15 K above the LCST. Taking into account the sensitivity of LSPR for the used nanoparticle arrays of $d\lambda_\perp/dn$=183 nm/RIU, such effect leads to a red shift of LSPR wavelength by about 20 nm. Same temperature change can also affect LSPR through other effects. Firstly, let us investigate the impact of the temperature-induced expansion of used glass substrate that results in modulating the nanoparticle arrays period Λ. As determined for the herein used geometry from our previous data(1), LSPR shifts with the period Λ by a factor $d\lambda_{LSPR}/d\Lambda$=0.6. As the herein used BK7 glass substrate exhibits the expansion coefficient of 7.1×10^{-6} K^{-1}, temperature increase of 15 K then leads to a change in the period of 0.045 nm translating negligible LSPR shift of 0.03 nm. Another important effect can be ascribed to temperature-driven changes in the refractive index changes of water and BK7 glass. For water and BK7 glass, the refractive index changes of $dn/dT = -1.2 \times 10^{-4}$ RIU/K(2) and 1.6×10^{-6} RIU/K at T = 32 °C (3), respectively. The temperature change of 15 K than translates to a shift LSPR by of -0.3 nm and 0.005 nm.

<u>3. Evaluation of the collapsing process for different heating pulse lengths</u>

The collapsing process was investigated for short (10 ms) and long (60 ms) optical heating pulses. The measured optical signal following the refractive index changes in the pNIPAAm-based polymer layer was fitted with single and two-exponential functions. The comparison of the respective fits is presented in Figure S2.

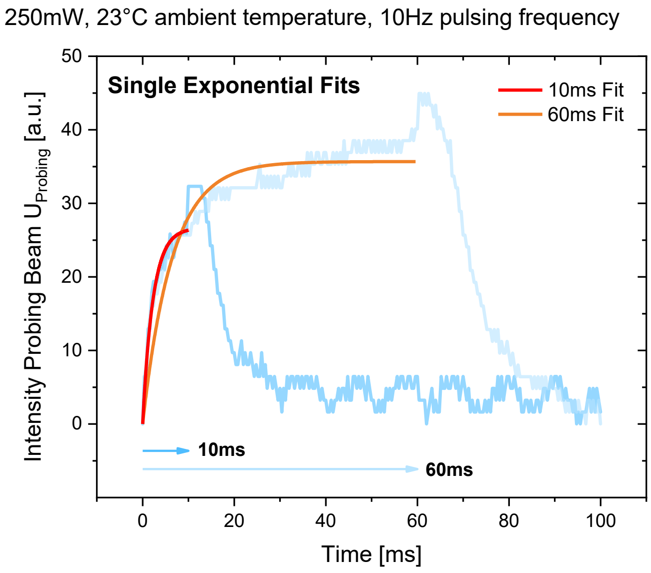

a)

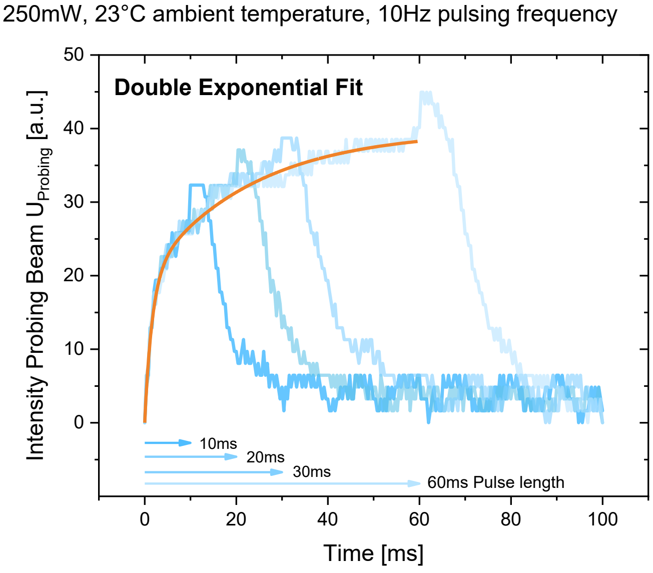

Figure S2 Fitting of the collapsing kinetics with a) single exponential function and b) two-exponential function.

<u>4. Collective heating regime</u>

Based on the theory of plasmonic heating on arrays of metallic nanoparticles summarized by Baffou et al.[(4)], there can be used the following dimensionless parameter ξ in order to distinguish between the regime when the individual nanoparticles are heated, or the collective heating of the overall irradiated area occurs:

$$\xi = \frac{\Lambda^2}{3La} \ , \tag{1}$$

where Λ is the period, L is the diameter of the irradiated area, and a is the nanoparticle diameter. Since for the herein used parameters (Λ=0.450 μm, L=30 μm, a=0.2-0.26 μm) the parameter is <0.1, the collective heating regime holds. Then the time constant with which the heating and cooling occurs can be estimated as:

$$\Delta t = \frac{L^2}{4\,\kappa/\rho c_p}, \tag{2}$$

where κ is thermal conductivity of water, ρ is the water density, and c_p is the heat capacity of water (yielding $\kappa/pc_p \sim 1.4\ 10^{-7}\ m^2 s^{-1}$. For the herein used diameter $L=30\ \mu m$, the response time $\Delta t \approx 0.4\ ms$ is predicted. [5]

The penetration depth of the heat transport into the surrounding medium upon periodic heating has been derived as

$$\delta_{th} = \sqrt{\frac{\kappa/pc_p}{\omega}},$$

where ω is the angular frequency of the heating beam power modulation.[5] For the frequency of 50 Hz, corresponding to a pulse width of 10 ms, the thermal penetration depth is estimated to be in the order of 30 μm. Using this relation to estimate the thermal penetration depth within the characteristic time Δt ($w_c = \pi/\Delta t$) yields $\delta_{th,c} \approx 6\ \mu m$. In both cases, the characteristic depth is much larger than the thickness of the swollen hydrogel layer (1μm) and we subsequently assume uniform temperature across the hydrogel layer.

5. Model for polymer networks swelling kinetics

The hydrogel swelling kinetics is described by using a displacement function of $u(x)$ that can be related to changes in the polymer volume fraction Φ as:

$$\Phi = \frac{\Phi_0}{1+\frac{\partial u}{\partial x}}, \tag{3}$$

where Φ_0 states for the polymer volume fraction in the equilibrium state. In general, the swelling and collapsing kinetics can be described by using the gel collective diffusion coefficient D that is proportional to the osmotic bulk modulus K depending on the osmotic pressure as:

$$K = \Phi \frac{\partial \Pi}{\partial \Phi}. \tag{4}$$

The osmotic pressure Π can be obtained from the Flory-like mean-field mixing energy and counteracting stress due to the elastic properties. In Flory Huggins theory, the mixing of the polymer with the solvent is taken into account by using the parameter χ, which depends on the miscibility of the polymer with the solvent and the respective osmotic pressure yields:

$$\Pi \propto \left(-\ln[1-\Phi]-\Phi-\chi\Phi^2\right). \tag{5}$$

Then, the dependence of collective diffusion coefficient on the Flory-Huggins parameter χ can be taken into account as:

$$D = A + B \cdot \Phi \chi \frac{\partial^2 \Pi}{\partial \Phi \partial \chi} = A + 2B\chi\Phi^2 = A + \frac{B'}{\left(1 + \frac{\partial u}{\partial x}\right)^2}, \quad (6)$$

where A, B' and B are constants.

6. Numerically solving of the model

The investigated geometry of a thin layer that is allowed to swell only in perpendicular direction to its surface is illustrated in Figure 6(a). The volumetric changes can be then described in one dimension with a time-dependent displacement distance $u(x,t)$, where the Cartesian axis x represents the allowed direction for swelling and collapse. The 1-D partial differential Fick-like diffusion equation:

$$\frac{\partial u(x,t)}{\partial t} = \frac{\partial}{\partial x}\left[D \frac{\partial u(x,t)}{\partial x}\right] \quad (7)$$

was used to describe the volumetric changes and it was solved using the 1D MATLAB PDE solver of the MATLAB software (Mathworks Inc. USA). This environment relies on the discretization of the space and time dimensions in order to numerically determine the solution $u(x,t)$.

The solution of this 1D parabolic partial differential equation (PDE) requires the definition of the initial state of the system, and of the boundary conditions for the displacement u and its first derivative of space. The initial condition is expressed as initial value for the displacement: $u(x,t=0)$ and it was set different for the swelling and collapsing processes, in order to take into account the direction of the displacement u. Moments before swelling, the thin layer is in a collapsed state and thus there was assumed a negative displacement along the x axis, and it possesses a polymer volume fraction larger than the equilibrium swollen state value $\Phi_0 = 1/\alpha$, where α is the polymer layer swelling ratio, set as 1.2 for this explanation, as this value allows for convergence of the model and clear visualization of the model's main features. This condition is expressed by $u(x,t=0) = -x(1-1/\alpha)$. On the other hand, before collapsing, the system is in a swollen state, corresponding to a positive displacement along the x axis, and it possesses a polymer volume fraction smaller than the equilibrium value Φ_0. This condition is expressed by $u(x,t=0) = x(\alpha-1)$. The imposed boundary conditions for the displacement $u(x=0,t)$ and for its first derivative of space $\frac{\partial u}{\partial x}(x=h_0,t)$, where $x=0$ corresponds to the inner interface and $x=h_0$ to the outer interface, are: the absence of displacement at the inner interface $u(x=0,t)=0$, as the layer is constrained to the substrate, and the absence of stress at the outer interface $\frac{\partial u}{\partial x}(x=h_0,t)=0$, as the forces at this interface are at equilibrium. The constants used in equation (6) are set to as $A=1, B'=-0.5$, and α=1.2 s, which allowed for a clear visualization of the discussed kinetics features.

7. Refractive index dependence on the swelling ratio of the hydrogel

The propagation of light through a mixture of two substances, a medium with permittivity ε_m with small inclusions ($diameter\ d_i \ll \lambda$) with ε_i can be described as the propagation through

a homogeneous medium with an effective refractive index ε_{eff} by the use of effective medium approximations.

Maxwell-Garnett derived an analytical expression for the refractive index of small metallic inclusions in an otherwise homogeneous medium. The model has also successfully been employed to describe dielectric inclusions as for hydrogels [(6)]. One of the prerequisites of this model, however, is the assumption of a small volume fraction Φ of inclusions ($\Phi \ll 1$). This could limit the applicability to fully collapsing hydrogel layers. The effective refractive index of a mixture of inclusions with ε_i in a medium with ε_m according to Maxwell Garnett is given by

$$\frac{\varepsilon_{eff} - \varepsilon_m}{\varepsilon_{eff} + 2\,\varepsilon_m} = \Phi \frac{\varepsilon_i - \varepsilon_m}{\varepsilon_i + 2\,\varepsilon_m}. \quad (1)$$

This can be solved analytically for the effective refractive index ε_{eff}

$$\varepsilon_{eff} = \varepsilon_m \frac{2\, f_i(\varepsilon_i - \varepsilon_m) + \ \varepsilon_i + 2\varepsilon_m}{2\varepsilon_m + \varepsilon_i - \Phi(\varepsilon_i - \varepsilon_m}. \quad (2)$$

Bruggeman addressed the problem of high volume content fraction by a symmetric treatment of the inclusion and the medium. The solution for a single type of inclusion with ε_i is given by

$$0 = \Phi \frac{\varepsilon_i - \varepsilon_{eff}}{\varepsilon_i + 2\,\varepsilon_{eff}} + (1 - \ \Phi) \frac{\varepsilon_m - \varepsilon_{eff}}{\varepsilon_m + 2\,\varepsilon_{eff}}. \quad (3)$$

Both approaches assume homogeneous field distribution throughout the medium, which is not fulfilled with a high density of metallic particles, cluster formation, or long-range ordering effects.

The theories are however suitable for the treatment of dielectric inclusions in dielectric media ($\varepsilon_i, \varepsilon_m \geq 0$).[(7)] For the materials considered in this work, hydrogel inclusions ($\varepsilon_i \approx 2.25$) in water ($\varepsilon_m = 1.774$) the difference in permittivity and is very small and the ratio of the permittivities $\varepsilon_i/\varepsilon_m$ is close to 1. In this regime, both approaches predict a linear dependence of the effective refractive index on the fill factor f, with R^2>99.95%, as shown in Figure S3.

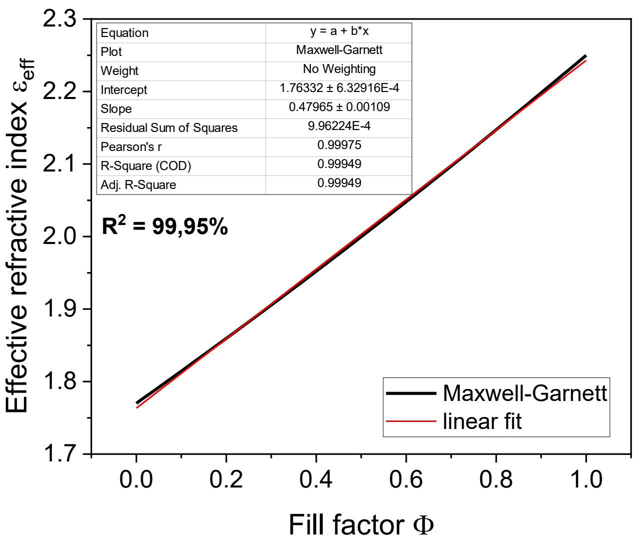

a)

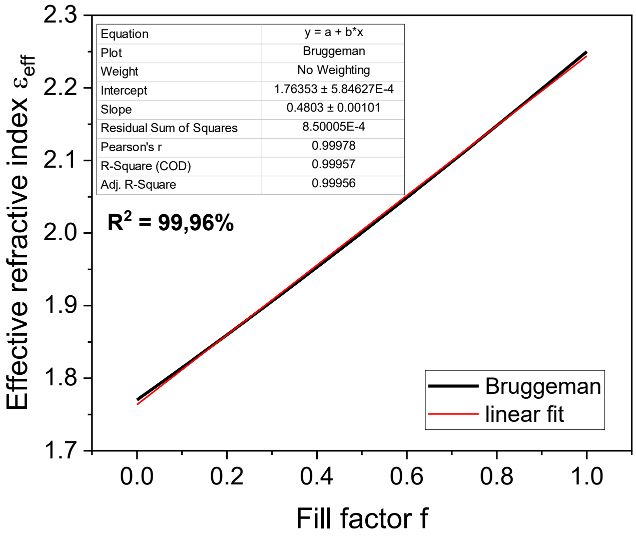

b)

Figure S3: Linear dependence of the effective refractive index on the fill factor f predicted by a) the Maxwell Garnett theory and b) the Bruggeman theory.

8. References

(1) Nestor G. Quilis, Mederic Lequeux, Pryiamvada Venugopalan, Imran Khan, Souhir Boujday, Wolfgang Knoll, Marc Lamy de la Chapelle, Jakub Dostalek, Tunable laser interference lithography preparation of plasmonic nanoparticle arrays tailored for SERS, Nanoscale, 2018, 10, 10268-10276

(2) Alexey N. Bashkatov , Elina A. Genina, Water refractive index in dependence on temperature and wavelength: a simple approximation, Saratov Fall Meeting 2002: Optical Technologies in Biophysics and Medicine IV, Valery V. Tuchin, Editor, Proceedings of SPIE Vol. 5068 (2003)

(3) Marion Englert, Peter Hartmann, and Steffen Reichel "Optical glass: refractive index change with wavelength and temperature", Proc. SPIE 9131, Optical Modelling and Design III, 91310H (1 May 2014);

(4) Baffou, G.; Berto, P.; Bermúdez Ureña, E.; Quidant, R.; Monneret, S.; Polleux, J.; Rigneault, H. Photoinduced Heating of Nanoparticle Arrays. *ACS Nano* **2013**, *7* (8), 6478–6488. https://doi.org/10.1021/NN401924N/SUPPL_FILE/NN401924N_SI_001.PDF

(5) P. Berto, M. Mohamed, H. Rigneault et al., Time-harmonic optical heating of plasmonic nanoparticles. Physical Review B - Condensed Matter and Materials Physics 2014, 90 (3). https://doi.org/10.1103/PhysRevB.90.035439

(6) Matthew M.Braun, LaurentPilon, Effective optical properties of non-absorbing nanoporous thin films, Thin Solid Films, Volume 496, Issue 2, 21 February 2006, Pages 505-514

(7) Matthias J. N. Junk, Ilke Anac, Bernhard Menges , Ulrich Jonas, Analysis of Optical Gradient Profiles during Temperature- and Salt-Dependent Swelling of Thin Responsive Hydrogel Films, Langmuir 2010, 26, 14, 12253–12259
